# Research on interpersonal violence in schizophrenia: based on different victim types

**DOI:** 10.1186/s12888-022-03820-7

**Published:** 2022-03-08

**Authors:** Yong He, Yan Gu, Meiling Yu, Yan Li, Gangqin Li, Zeqing Hu

**Affiliations:** Department of Forensic Psychiatry, West China School of Basic Medical Sciences and Forensic Medicine, Sichuan University, Sichuan 610041 Chengdu, China

**Keywords:** Schizophrenia, Interpersonal violence, Victim relationship, Criminological characteristics, Clinical characteristics, Demographic characteristics

## Abstract

**Background:**

Schizophrenia is one of the most common severe mental disorders associated with an increased risk of violence. The present study compares the demographical, clinical, and criminological characteristics of the patients with schizophrenia who committed different types of violence to relatives, acquaintances, or strangers.

**Method:**

Archives of the violent offenders with schizophrenia referred to forensic psychiatric assessments from January 2015 to December 2019 in the West China Forensic Medicine Assessment Center in China were analyzed. The demographic information, mental illness history, and criminological characteristics of the offenders were collected. The clinical symptoms, previous violent behaviors, and social deficits were also evaluated. One-way ANOVA, Kruskal–Wallis test, Chi-square test, and logistic regression analysis were enrolled to do the statistical analysis.

**Results:**

The study enrolled 332 cases: 165 cases (49.7%) in the acquaintance victim group (AV), 96 cases (28.9%) in the relative victim group (RV), and 71 cases (21.4%) in the stranger victim group (SV). The multinomial logistic regression analysis revealed that older patients were less likely to attack relatives (OR = 0.966, 95% CI = 0.944–0.990; *p* = 0.005), and strangers, (OR = 0.971, 95% CI = 0.944–0.998; *p* = 0.034). Patients who lived with others were more inclined to attack relatives (OR = 15.057, 95% CI = 3.508–64.628; *p* < 0.001). Additionally, employed patients were more likely to attack strangers (OR = 2.034, 95% CI = 1.036–3.994; *p* = 0.039). The regression equation did not include psychiatric symptoms. For RV and AV victims, the risk of death was higher compared to that of SV victims (OR = 13.778, *p* < 0.001; OR = 2.663, *p* = 0.014).

**Conclusion:**

In the interpersonal violence cases committed by schizophrenia patients, the victim type correlates with demographic characteristics of offenders such as living situation, age, and employment status, but not with the psychiatric symptoms. The majority of victims were acquaintances and relatives, and the relative victims having more severe injuries. In order to decrease interpersonal violence, especially violent crimes, more people, especially family members and neighbors, should be educated about symptoms of schizophrenia, the ways to communicate with the patients, and the methods for crisis management.

## Introduction

Schizophrenia is a severe mental disorder [1], which the public often related to violent behavior [2, 3], and several studies have shown that schizophrenia increases the risk of violent behavior [4–7]. Previous studies have focused on the clinical and criminological characteristics of schizophrenia patients committing violent acts, most of whose control groups were healthy people or patients without violent behavior. Few studies have sought to compare the characteristics of schizophrenic patients who commit interpersonal violence with different victim types. The generation of interpersonal violence is an interactive process of the perpetrator and victim, relevant to the surrounding people and the environment. The course and the outcome of violent behavior from schizophrenia may vary depending on the type of victim, which may elicit different social impacts. Compared to clinical management of the mentally ill (eg. risk assessment, improved treatment), the relatively rare and unpredictable violence among psychiatric patients are more likely to raise public concerns and increase the stigma of the patients [8].

Cases of injury or murder among strangers in public places tend to raise public awareness and panic. Previous studies have also documented several forms of victimization. The investigation of homicide cases has shown that the mentally ill are less likely to attack strangers [9–13]. In contrast, families and friends of individuals with mental disorders face the most significant risk of harm [14]. Patients with severe mental disorders like schizophrenia commit half of the violent acts directly on family members [15, 16]. A Japanese study showed that 34.8% of family members of schizophrenia patients had experienced physical violence from the patient [17]. According to a meta-analysis, at least 40% of relatives of mental disordered people were injured by the patients [18]. Schizophrenia has a stronger association with homicide than other diagnoses of mental disorders [19, 20]. Among patients with mental disorders who have murdered relatives, more than 50% were schizophrenia patients [21, 22]. Concerning the injury severity, relatives of people with mental disorders had a higher probability of suffering severe or fatal violence [23, 24]. Psychiatric criminals have a higher propensity to commit crimes alone, in public, and against strangers than nonpsychiatric criminals [25].

The study of the risk factors related to offenders in different categories of victim is insufficient. How do demographic characteristics and clinical characteristics influence the type of victims of violence in people with schizophrenia? Moreover, what are the criminological characteristics of the offenders according to different types of victims? To answer these questions, we conducted a comparative study of forensic psychiatry assessed cases of schizophrenia suspected of violent crimes based on the categories of the victim hoping to provide a framework for identifying individuals at high risk of violent victimization and developing preventive measures.

### Study subjects

We reviewed archival data of schizophrenia suspects assessed at the West China Forensic Medicine Assessment Center in Sichuan Province, China, from January 2015 to December 2019, with the inclusion criteria as following: (1) have interpersonal violence; (2) was diagnosed with schizophrenia according to the criteria of the Chinese Classification of Mental Disorders-3^rd^ edition (CCMD-3), and no other comorbidities; (3) Completed case data. In this study the interpersonal violence was about physical violence against others’ bodies. All the eligible cases were categorized into 3 groups according to different types of victims(relatives, acquaintances, or strangers as victims respectively).If there were multiple victims in one case, we grouped them based on the first victim. The ethics committee board of Sichuan University approved this study.

## Methods

This retrospective study used a framework of forensic psychiatry sample, with the required law enforcement agencies providing the necessary information for the assessment, including demographic information, medical records, and the files of criminals. The data from their forensic psychiatric assessment and the files of criminals contain demographic, gender, ethnicity, year of birth, education level, marital status, place of residence, employment history, living situation, family history of mental illness, and drug abuse. We collected the data of criminological characteristics, the location of the interpersonal violence, the gender and relationship of the victims, the tools used, and the patients' criminal history. We applied the Brief Psychiatric Rating Scale to evaluate clinical characteristics and psychiatric symptoms, the Social Disability Screening Schedule (SDSS) to appraise the social function. We also collected the patient's past hospital visits and medication status. In the forensic psychiatric assessment center, two psychiatrists evaluated the patient's mental status according to the CCMD-3, which originated in the ninth edition of the International Classification of Diseases [26, 27], but now corresponds to the 10th edition of the International Classification of Diseases [28].

### Measurement

#### The Brief Psychiatric Rating Scale (BPRS)

The Brief Psychiatric Rating Scale (BPRS) [29] is one of the standard instruments commonly used in daily practice to evaluate the severity of schizophrenia-related symptoms, and the 18-item scale is widely used [30]. This scale includes five subscales as follows: Affect (anxiety, guilt, depression, somatic); Positive Symptoms (thought content, conceptual disorganization, hallucinatory behavior, grandiosity); Negative Symptoms (blunted affect, emotional withdrawal, motor retardation); Resistance (hostility, uncooperativeness, suspiciousness); and Activation (excitement, tension, mannerisms-posturing) [31].

#### The Social Disability Screening Schedule(SDSS)

The Social Disability Screening Schedule (SDSS) used in this study was a simplified Chinese version of the World Health Organization's Disability Assessment Schedule [32], which assesses ten different aspects of social function, and each item has a score ranging from 0 to 2. The higher the score, the worse the social function outcome. Twelve areas epidemiological survey of mental disorders in China stipulated a total score of ≥ 2 points, which indicates a functional social deficit [33].

### Statistical analysis

All analyses were performed using IBM SPSS Statistics (version 20.0). The significance level was 0.05 (2-sided). Three independent groups were compared using a one-way analysis of variance (ANOVA) or the Kruskal–Wallis H test. The Chi-squared test was used to compare the categorical variables across the three groups. Bonferroni’s correction for multiple comparisons was used for pairwise comparisons between groups. Multinomial logistic regression was used to analyze the risk factors for offenders targeting different type of victims.

## Results

A total of 332 cases were included in the current study, while 165 cases (49.7%) in the Acquaintances Victim group (AV), 96 cases (28.9%) in the Relatives Victim group (RV), and 71 cases (21.4%) in the Stranger Victim group (SV). The average age of the offenders was 40.16 ± 12.04 years old, 97.0% were Chinese Han population, and 81.0% were men. There were 79.2% of the offenders had a low level of education (≤ 9 years), most of them lived in rural areas (73.2%), and 23.5% of them never had a job before. There were 79.2% of the patients were unemployed at the time of the violence. Regarding marriage, only 36.4% were married, while the rest were unmarried, divorced, or widowed. In terms of the living situation, 79.2% lived with others and 20.8% lived alone. A total of 73.8% had sought treatment in a psychiatric facility before the violence occurred, and 55.1% were hospitalized. The majority of patients (67.4%) had the disease for more than five years. Nearly four-fifths of the patients did not take medicine at the time of committing the violence. A family history of mental illness is present in 8.7% of cases. The percentage of patients with a criminal record was 9.3%. Regarding substance use, 12.3% of offenders drank alcohol before committing the interpersonal violence, and none were drug users (Table 1).

**Table 1:** Demographic, clinical and criminological characteristics of the study sample

	*n* (%)
**Demographic characteristics**	
**Age [Mean (SD)]**	40.16(12.04)
**Sex**	
Male	269(81.0%)
Female	63(19.0%)
**Ethnicity**	
Han	322(97.0%)
Others	10(3.0%)
**Education years**^a^	
≤ 9	259(79.2%)
> 9	68(20.8%)
**Living in rural areas**	
Yes	243(73.2%)
No	89(26.8%)
**Ever been employed**	
Yes	254(76.5%)
No	78(23.5%)
**Employment status**	
Employed	69(20.8%)
Unemployed	263(79.2%)
**Marital status**	
Married	121(36.4%)
Others^c^	211(63.6%)
**Living situation**	
Living with others	263(79.2%)
Living alone	69(20.8%)
**Clinical characteristics**	
**History of psychiatric treatment**	
Yes	245(73.8%)
No	87(26.2%)
**Hospitalization**	
Yes	183(55.1%)
No	149(44.9%)
**Illness duration(years)**^b^	
< 5	108(32.6%)
≥ 5	223(67.4%)
**Medication at the time of violence**	
Yes	69(20.8%)
No	263(79.2%)
**Alcohol at the time of violence**	
Yes	41(12.3%)
No	291(87.7%)
**Drug users**	
Yes	0(0.00%)
No	332(100.0%)
**Family history of mental illness**	
Yes	29(8.7%)
No	303(91.3%)
**Criminological characteristics**	
**History of criminal offense**	
Yes	31(9.3%)
No	301(90.7%)
**Interpersonal violent types**	
Physical assault	217(65.4%)
Murder	115(34.6%)
**The location of the crime**	
Co-residence	77(23.2%)
Residence of offender	20(6.0%)
Residence of victim	56(16.9%)
Public place	173(52.1%)
Remote place	6(1.8%)
**The use of tools**	
Without tools	54(16.3%)
Bring own tools	134(40.4%)
Tools on-site	144(43.4%)
**Number of attacks**	
Single	51(15.4%)
Repeated	281(84.6%)
**The characteristics of victims**	
**Sex**	
With female	137(41.3%)
Male	195(58.7%)
**Death**	
Yes	119(35.8%)
No	213(64.2%)

^a^There were five missing data

^b^There was one missing data

^c^Others: unmarried, divorced, widowed

For the types of interpersonal violence, 217 cases (65.4%) were physical assault, and 115 cases (34,6%) were murder. In terms of the location of the interpersonal violence, public places accounted for the highest proportion at 52.1%, followed by co-residence 23.2%, and victim residence (16.9%); 83.7% of suspected offenders used tools, of which 48.2% brought tools to the criminal scene, and 51.8% used tools on-site. There were 84.6% offenders had repeated attack behavior. In 41.3% of the cases, the victims contain at least one woman, and in 35.8% of the cases, the victims encountered death problems (Table 1).

### Comparison of characteristics among groups

#### Demographic characteristics

Gender, ethnicity, education level, and employment history did not differ among the groups (*p* > 0.05). In addition, differences among the three groups were found based on demographic factors such as age, living situation, employment status, place of residence, and marital status. The age of the AV (42.98 ± 12.75 years old) was significantly higher than that of the RV (37.07 ± 11.36 years old) and the SV (37.79 ± 9.56 years old) statistically (*p* < 0.001). By living situation, more patients lived with others in the RV (97.9%) group than the AV (72.1%) and SV (70.4%) (*p* < 0.001). Regarding the employment status, more patients in the SV group were in employment compared to those in the AV (17.0%) and RV (17.7%) (*p* = 0.009). A marginal significant difference was found in place of residence categories (*p* = 0.056) and marital status (*p* = 0.050), that a higher percentage of schizophrenia offenders attacking acquaintances living in the rural area (78.8%) compared to those in the RV (69.8%) and SV (64.8%). The proportion of married patients in the SV (28.2%) was lower than that of the AV (34.5%) and RV (45.8%) (*p* = 0.050), (Table 2).

**Table 2:** Comparison of demographic, clinical and criminological characteristics among groups

	RV *n* = 96	AV *n* = 165	SV *n* = 71	F/x^2^	*p*	Post hoc analysis
**Demographic characteristics**						
**Age [Mean (SD)]**	37.07(11.36)	42.98(12.75)	37.79(9.56)	9.541	** < 0.001**	AV > SV = RV
**Sex**						
Male	76(79.2%)	131(79.4%)	62(87.3%)	2.333	0.311	
Female	20(20.8%)	34(20.6%)	9(12.7%)			
**Ethnicity**						
Han	93(96.9%)	159(96.4%)	70(98.6%)	0.689	0.839*	
Others	3(3.1%)	6(3.6%)	1(1.4%)			
**Education years**^a^						
≤ 9	76(80.9%)	127(77.9%)	56(79.2%)	0.346	0.841	
> 9	18(19.1%)	36(22.1%)	14(20.0%)			
**Living in rural areas**						
Yes	67(69.8%)	130(78.8%)	46(64.8%)	5.754	0.056	
No	29(30.2%)	35(21.2%)	25(35.2%)			
**Ever been employed**						
Yes	72(75.0%)	122(73.9%)	60(84.5%)	3.255	0.196	
No	24(25.0%)	43(26.1%)	11(15.5%)			
**Employment status**						
Employed	17(17.7%)	28(17.0%)	24(33.8%)	9.319	**0.009**	SV > AV
Unemployed	79(82.3%)	137(83.0%)	47(66.2%)			
**Marital status**						
Married	44(45.8%)	57(34.5%)	20(28.2%)	6.010	0.050	
Others^c^	52(54.2%)	108(65.5%)	51(71.8%)			
**Living situation**						
Living with others	94(97.9%)	119(72.1%)	50(70.4%)	28.771	** < 0.001**	RV > AV = SV
Living alone	2(2.1%)	46(27.9%)	21(29.6%)			
**Clinical characteristics**						
**History of psychiatric treatment**						
Yes	78(81.2%)	119(72.1%)	48(67.6%)	4.405	0.111	
No	18(18.8%)	46(27.9%)	23(32.4%)			
**Hospitalization**						
Yes	57(59.4%)	85(51.5%)	41(57.7%)	1.767	0.413	
No	39(40.6%)	80(48.5%)	30(42.3%)			
**Illness duration(years)**^b^						
< 5	32(33.3%)	53(32.1%)	23(32.9%)	0.043	0.979	
≥ 5	64(66.7%)	112(67.9%)	47(67.1%)			
**Medication at the time of violence**						
Yes	75(78.1%)	136(82.4%)	52(73.2%)	2.641	0.267	
No	21(21.9%)	29(17.6%)	19(26.8%)			
**Alcohol at the time of violence**						
Yes	7(7.3%)	25(15.2%)	9(12.7%)	3.473	0.176	
No	89(92.7%)	140(84.8%)	62(87.3%)			
**Family history of mental illness**						
Yes	13(13.5%)	10(6.1%)	6(8.5%)	4.270	0.118	
No	83(86.5%)	155(93.9%)	65(91.5%)			
**Criminological characteristics**						
**History of criminal offense**						
Yes	5(5.2%)	15(9.1%)	11(15.5%)	5.123	0.077	
No	91(94.8%)	150(90.9%)	60(84.5%)			
**Interpersonal violent types**						
Murder	63(65.6%)	43(26.1%)	9(12.7%)	61.202	** < 0.001**	RV > AV = SV
Physical assault	33(34.4%)	122(73.9%)	62(87.3%)			
**The location of the interpersonal violence**						
Co-residence	70(72.9%)	7(4.2%)	0(0.0%)	222.041	< 0.001*	RV > AV = SV
Residence of offender	2(2.1%)	15(9.1%)	3(4.2%)			-
Residence of victim	7(7.3%)	48(29.1%)	1(1.4%)			AV > RV = SV
Public place	16(16.7%)	94(57.0%)	63(88.7%)			SV > AV > RV
Remote place	1(1.0%)	1(0.6%)	4(5.6%)			SV > AV
**The use of tools**						
Without tools	9(9.4%)	23(13.9%)	22(31.0%)	65.444	** < 0.001**	SV > AV = RV
Bring own tools	16(16.7%)	81(49.1%)	37(52.1%)			SV = AV > RV
Tools on-site	71(74.0%)	61(37.0%)	12(16.9%)			RV > AV > SV
**Number of attacks**						
Single	12(12.5%)	24(14.5%)	15(21.1%)	2.504	0.286	
Repeated	84(87.5%)	141(85.5%)	56(78.9%)			
**The characteristics of victims**						
**Sex**						
With female	57(59.4%)	61(37.0%)	19(26.8%)	20.409	** < 0.001**	RV > AV = SV
Male	39(40.6%)	104(63.0%)	52(73.2%)			
**Death**						
Yes	64(66.7%)	46(27.9%)	9(12.7%)	60.785	** < 0.001**	RV > AV > SV
No	32(33.3%)	119(72.1%)	62(87.3%)			

*Note*: *SD* Standard Deviation, *RV* Relatives Victim group, *AV* Acquaintances Victim group, *SV* Stranger Victim group

^*^Fisher exact test

^a^There were two missing data in the RV and AV, and one data missing in the SV

^b^There was one missing data in the SV

^c^Others: unmarried, divorced, widowed

#### Clinical characteristics

In concern of the clinical characteristics, there were no statistically significant differences in terms of the history of psychiatric treatment, hospitalization, illness duration, treatment, and alcohol use when committing the index interpersonal violence, as well as family history of mental disorders (*p* > 0.05)(Table 2).

Regarding the social function, the median score of SDSS for offenders in RV was 8.00, in AV was 8.00, and in SV was 7.00, and the score distributions did not differ significantly among the three groups (H = 4.075, *p* = 0.130) (Table 3).

**Table 3:** Results of SDSS and BPRS

		Groups			ANOVA/Kruskal–Wallis test		Post hoc analysis		
		RV	AV	SV			RV-AV	RV-SV	AV-SV
		*n* = 96	*n* = 165	*n* = 71	*H/F*	*p*	*△p*	*△p*	*△p*
**SDSS**	Median (25%,75%)	8.00(6.00,11.00)	8.00(5.50,11.00)	7.00(3.00,11.00)	4.075	0.130	-	-	-
**Affect**	Median (25%,75%)	9.00(8.00,11.00)	9.00(8.00,11.00)	9.00(7.00,10.00)	2.090	0.352	-	-	-
**Positive**	Median (25%,75%)	12.00(10.00,15.00)	13.00(10.00,15.50)	10.00(8.00,14.00)	9.604	0.008	1.000	0.026	0.010
**Negative**	Median (25%,75%)	6.00(4.00,8.00)	6.00(4.50,7.00)	5.00(4.00,6.00)	13.026	0.001	1.000	0.003	0.004
**Activation**	Median (25%,75%)	5.00(4.00,6.75)	5.00(4.00,6.00)	5.00(4.00,6.00)	0.457	0.796	-	-	-
**Resistance**	Median (25%,75%)	10.00(8.25,11.00)	10.00(8.00,12.00)	8.00(6.00,11.00)	12.571	0.002	1.000	0.006	0.003
**BPRS-Total**^a^	Mean (SD)	45.68(9.481)	44.74(9.302)	40.38(9.232)	7.386	0.001	1.000	0.001	0.003

^a^one-way ANOVA

*△p*: adjusted *P*

*SD* Standard Deviation

*RV* Relatives Victim group, *AV* Acquaintances Victim group, *VS* Stranger Victim group

The BPRS indicated no significant differences in the affect and activation scores among groups (*p* > 0.05), however, it showed statistically significant differences in positive symptoms (H = 9.604, *p* = 0.008), negative symptoms (H = 13.026, *p* = 0.001), resistance (H = 12.571, *p* = 0.002), and the total score (F = 7.386, *p* = 0.001) among the three groups. Pairwise comparison after Bonferroni post hoc test found that the median scores of positive symptoms, negative symptoms, and resistance were significantly lower in the SV group than the other two groups (*p* < 0.05); there was no statistically significant difference in psychiatric symptom scores between the AV group and the RV group (*p* > 0.05) (Table 3).

#### Criminological characteristics

There was a higher proportion (15.5%) of offenders in the SV who had previous criminal history than that in the RV (5.2%) and AV (9.1%) (*p* = 0.077). And there was a significant difference regarding the interpersonal violence location and types among the three groups (*p* < 0.001). Murders were more likely to occur among relatives (65.6%), while physical assault in acquaintances (73.9%) and strangers (87.3%). Violence targeting relatives occurred more often in the co-residence of the perpetrator and the victim (72.9%), while attacking acquaintances occurred more often in public places (57.0%) and the victims’ residence (29.1%). Cases in which the victims were strangers occurred more often in public places (88.7%). Regarding the gender of victims, 41.3% of cases involved female victims, and the difference among the RV (59.4%), the AV (37.0%), and SV (26.8%) was statistically significant (*p* < 0.001). In terms of the severity, victims deceased in 35.8% of cases. The RV had the highest death rate of 66.7%, followed by the AV (27.9%), and the lowest was the SV (12.7%) (*p* < 0.001). Pairwise comparison after Bonferroni post hoc test also showed that the differences between the three groups were statistically significant. There were 83.7% of the offenders used tools, among which the RV used tools on-site in the highest proportion, accounting for 74.0%, while the other two groups’ patients brought their tools were 49.1% and 52.1%, respectively, accounted for the highest percentage (Table 2). About specific victim relationships, in the RV, the parent (32.3%) had the highest proportion, followed by the spouse (24.0%); in the AV, the neighbor (73.3%) had the highest percentage. For the occurrence of death, the death risk of the RV was 13.78 times(95% CI 6.081, 31.215, *p* < 0.001) higher than that in the SV; when it comes to the AV, the risk of death was 2.66 times (95%CI 1.224, 5.795, *p* = 0.014) higher than that of the SV.

#### Multinomial logistic regression analysis

To assess the risk factors of different victim types, we used multinomial logistic regression to analyze the demographic characteristics and clinical characteristics analysis with *p* < 0.05. The multinomial logistic regression analysis taking the AV group as a reference revealed that older patients had a lower possibility of attacking relatives, OR = 0.966, 95% CI = 0.9440.990, *p* = 0.005, and strangers, OR = 0.971, 95% CI = 0.9440.998, *p* = 0.034. This analysis also revealed that patients who lived with others were more likely to attack relatives, OR = 15.057, 95% CI = 3.508 - 64.628, *p* < 0.001, Furthermore, patients who were employed were more likely to attack strangers OR = 2.034, 95% CI = 1.036- 3.994, *p* = 0.039. The regression equation did not include psychiatric symptoms (Table 4).

**Table 4:** Multinomial logistic regression

group	factors	*p*	OR	95%CI
RV	Age	**0.005**	0.966	0.944–0.990
	Negative symptoms	0.637	1.023	0.931–1.124
	Positive symptoms	0.362	1.039	0.957–1.127
	Resistance	0.853	0.988	0.868–1.124
	Employed	0.952	0.979	0.488–1.963
	unemployed	Ref		
	Live with others	** < 0.001**	15.057	3.508–64.628
	Live alone	Ref		
SV	Age	**0.034**	0.971	0.944–0.998
	Negative symptoms	0.081	0.885	0.772–1.015
	Positive symptoms	0.490	0.965	0.873–1.067
	Resistance	0.186	0.908	0.786–1.048
	Employed	**0.039**	2.034	1.036–3.994
	Unemployed	Ref		
	Living with others	0.576	0.822	0.413–1.635
	Living alone	Ref		

*Note*: *RV* Relatives Victimization group, *AV* Acquaintances Victim group, *VS* Stranger Victim group, the reference group is AV

## Discussion

In our study, the highest percentage of victims was acquaintances (49.7%), followed by relatives (28.9%) and strangers (21.4%). The proportion of relatives was lower than that reported in two previous studies, whereas the rate of relatives was 69.4% and 43.1%, respectively [15, 16], while higher than that of a study in Sweden with 13% [24], and comparable to Morgan with 33.3% [25]. Different characteristic of offenders may result in the difference of victim targets. Some relatives of the schizophrenic patients may consider violence as an inevitable consequence of the disorder and tolerate the patient's violent behavior [34]. Generally, the victims would not report to the police unless being severely injured. However, the patient would usually be punished by law when committing violent acts against people outside the family. This may be one of the reasons why the data based on police records differ from the results of community surveys. In a Swedish study [24], only 13% of victims were relatives, while strangers made up 55.8%. In our study, strangers accounted for only 21.4%. This difference may also be from the study sample, in our study, we only focus on the original victim that was the first one being violently attacked. Police, security officers, and other people who were victimized in order to prevent the consequences of serious injuries were excluded, however, these kinds of victims were categorized as strangers in the aforementioned Swedish study [24].

In this study, the BPRS scores on positive symptoms, negative symptoms, and resistance symptoms of the offenders attacking strangers were lower than those attacking relatives and acquaintance. However, the multinomial regression analysis did not find significant contribution of these symptoms to the different types of victims, which suggesting that mental symptoms may not be the primary determinant of the type of victim.

A survey on schizophrenia patients who harm relatives and strangers found that insults, threats, and forced hospitalization were the primary inducement for the violence [35]. Patients who kill strangers are more likely to be homeless, exhibit anti-social behavior, and have fewer negative symptoms than those who kill family members [12].

Psychotic symptoms and violence have been associated for a long time, but the specific mechanism remains a mystery. Researchers have found that persecuted delusions can contribute to violent behavior in patients with schizophrenia [6], and delusions or hallucinations are related to violent behavior [36, 37]. However, studies have shown that delusions do not increase the patient’s overall violence risk, although they may impact individual patients’ violent behavior [38]. Delusional behavior does not typically lead to violent behavior [39], and rarely does the patient kills the victim by obeying the commanded auditory hallucination [35, 40]. The occurrence of violent behavior may be a maladaptive resolution of conflict by the patient [41] or enhanced response to the stress in the stimulus situation [42]. The chances of people exhibiting aggressive behavior are higher when they feel scared [43].

Patients with schizophrenia frequently have concomitant cognitive impairment [1, 44]. Studies have shown that executive dysfunction is related to violent behavior [45, 46], because of the impairment of ability to adapt to the environment. It is sometimes complicated for individuals to adapt their behavior to environmental changes and to have inadequate inhibition, resulting in maladaptive challenges in social settings and more violent responses [46]. Unable to deal with conflicts reasonably, the presence of psychotic symptoms such as delusions and hallucinations may simply increase the probability of a patient experiencing conflict, however, whether violent behavior will eventually occur, is associated with the patient's executive function and other cognitive abilities to assess and implement a specific conflict processing strategy.

Taken together, symptoms, perpetrator-victim relationships, and circumstances may interact in complex ways to lead to violence. According to the Situation Action Theory (SAT) [47, 48], the act of crime results from a perception-choice process of persons’ crime propensity and criminogenic exposure, and SAT maintains that acts of crime are best explained as moral actions. And a person’s crime propensity was a consequence of their morality and ability to exercise self-control. The influence of a setting’s moral context on action is always a question of its perceived moral context. A person’s criminogenic exposure may be seen as his or her encounters with settings in which the (perceived) moral norms and their (perceived) levels of enforcement (or lack of enforcement) encourage reaches of rules of conduct (stated in law) in response to particular opportunities or frictions in their daily life [48]. Suffering from schizophrenia may influence the patients’ morality and ability to exercise self-control and accurate perception of moral norms and enforcement in settings.

The multinomial regression analysis showed that employed and younger patients were more likely to attack strangers, and the younger patients living with others were more likely to attack relatives. The age, living situation, and employment may affect the living circumstances and the opportunity to connect with others. The stranger group of employed offenders can participate in more social life activities, and these activities reach out to more people than the other two groups, so it is more likely to contact strangers. Furthermore, they were less often co-live with others, lacking reasonable and adequate care and supervision. In the case of a patient confined at home, we assume a low risk of him or her harm a stranger.

The RV was more likely to live with others, and about 72.9% of the RV cases occurred at their co-residence, which suggests that most of the patients who attacked relatives were at home and had a small personal social circle, and the reason may be that they can only contact the relative who is caregivers at the most of the time.

The RV has a high proportion of cases involving female victims, which is consistent with previous studies showing that women account for most family victims [14, 15, 24] because caregivers of the patients were mainly spouses or mothers. In a survey of schizophrenia patients with parricide, 98.1% lived with their parents [35]. Patients with schizophrenia may have some behavioral disorders due to the impact of the disease. Parents and other co-residents would ask them to do things such as not hanging out at night, not smoking at night, cleaning their room, regularly eating, taking medication, and saving [35]. The patient may perceive these restricted behaviors as parental intimidation and hostility toward them [49]. However, as the main caregiver, mothers play a significant role in compulsory drug feeding, forced hospitalization, and providing discipline. As a result, conflicts between caregivers and patients occurred, and patients would attack co-living people. Patients often inflict violence on their victims before their homicide occurred, with 40.7% of patients violently abusing their victims before the homicide occurred [35].Regarding the severity of victimization, the incidence of death in the relative group exceeded that of the other two groups significantly. Previous studies have also shown that patients with mental disorders inflict more severe injuries on their relatives [24]. A possible explanation would be the location of the incident, considering that most cases occurred in the co-residence. It was easier for the offender to obtain tools such as knives and sticks because the offender was familiar with the area, and the severity of the attack was more significant than if the offender had no tools. Outsiders cannot prevent violence and treat victims because of the relatively secretive environment of the incident, which increases the risk of death for the victim. Furthermore, since the study sample was drawn from physical assault and murder cases of schizophrenic patients identified by public security organs, some less severe injuries in the relative victims possibly were not included in the study sample.

People’s movement patterns are determined by their individual routine activities and that people offend is related to their activity fields [48]. Frictions with their caregivers in daily life may also influence the patient’s criminogenic exposure. In China, family members are primarily responsible for supervising patients with mental disorders [50, 51]. However, the majority of family members lack the knowledge and ability to coping violent crisis of mentally ill [51]. Many patients have recurrent symptoms due to inadequate or incorrect treatment [52, 53]. As a result of residual symptoms [54], impaired cognitive and emotional function [44, 55], or stigma [56, 57], these patients repeatedly committed violent crimes. In this condition, the basic knowledge of mental disorders and violence prevention skills for family caregivers should be strengthened, which is crucial to disease rehabilitation and violence prevention. In the meanwhile, the community and government should make effort to construct a long-term sensible and reasonable management system for patients with mental disorders to improve their rehabilitation.

### Limitations

There are some limitations need to be addressed in the current study. First, we only included cases from one forensic medicine assessment center in Sichuan province. Although this forensic center is responsible for most of the forensic psychiatric assessments for offenders with mental disorder in Sichuan Province (home to over 80 million people) [58], the findings may not be generalized to the entire country or other regions. However, the analytic methodology demonstrated in this study could be adopted for analysis in other regions. Second, due to the limitation of retrospective study, some information of the offenders such as the education level data were missing.

## Conclusion

In the interpersonal violence cases committed by schizophrenia patients, the victim type correlates with demographic characteristics of offenders such as living situation, age, and employment status, but not with the psychiatric symptoms. Acquaintances and relatives are more likely to be injured by schizophrenic patients, Co-residents, caregivers and relatives were more vulnerable to suffer severe violence, especially the females.

It is essential to establish a guardianship system for patients with schizophrenia to improve caregiver awareness of the disease and risk management methods.

## Data Availability

The data that support the findings of this study will be available from the corresponding author on reasonable request. The data are not publicly available due to the sensitivity of the subjects.

## Abbreviations

CCMD-3: The third edition of the Chinese Classification of Mental Disorder
RV: Relatives victim group
AV: Acquaintances victim group
VS: Stranger victim group
BPRS: The Brief Psychiatric Rating Scale
SDSS: The Social Disability Screening Schedule
SAT: Situation Action Theory
